# *QuickStats:* Age-Adjusted Drug Overdose Death Rates,*^,^^†^ by State — National Vital Statistics System, United States, 2021

**DOI:** 10.15585/mmwr.mm7211a7

**Published:** 2023-03-17

**Figure:**
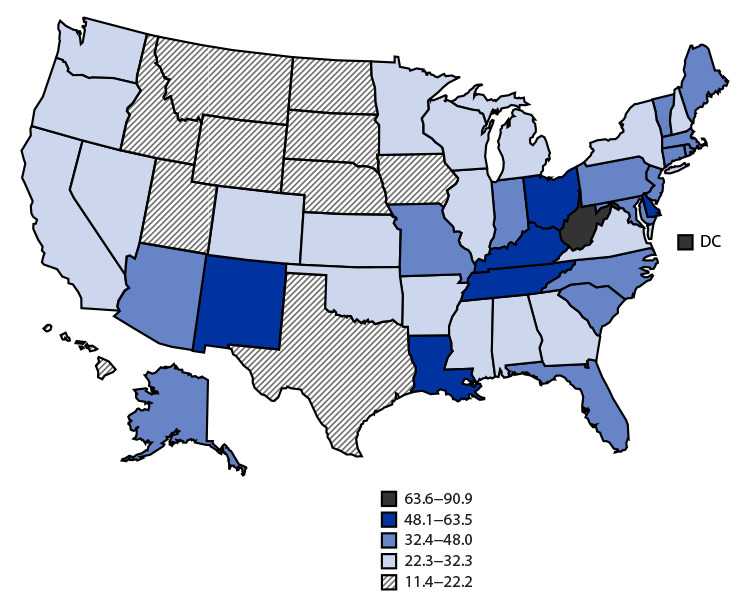
In 2021, the U.S. age-adjusted drug overdose death rate was 32.4 per 100,000 population. The highest rates were in West Virginia (90.9) and the District of Columbia (63.6); the lowest rates were in the Upper Midwest and Texas. The lowest state rates were those in Nebraska (11.4), South Dakota (12.6), and Iowa (15.3).

**Source**: National Center for Health Statistics, National Vital Statistics System, Mortality Data. https://www.cdc.gov/nchs/nvss/deaths.htm

For more information on this topic, CDC recommends the following link: https://www.cdc.gov/drugoverdose/index.html

